# A self-organizing, living library of time-series data

**DOI:** 10.1038/s41597-020-0553-0

**Published:** 2020-07-07

**Authors:** Ben D. Fulcher, Carl H. Lubba, Sarab S. Sethi, Nick S. Jones

**Affiliations:** 1 School of Physics, The University of Sydney, Sydney, NSW 2006 Australia; 2 Mathematics Department, Imperial College London, Huxley Building, Queen’s Gate, London SW7 2AZ UK

**Keywords:** Data mining, Databases

## Abstract

Time-series data are measured across the sciences, from astronomy to biomedicine, but meaningful cross-disciplinary interactions are limited by the challenge of identifying fruitful connections. Here we introduce the web platform, *CompEngine*, a self-organizing, living library of time-series data, that lowers the barrier to forming meaningful interdisciplinary connections between time series. Using a canonical feature-based representation, *CompEngine* places all time series in a common feature space, regardless of their origin, allowing users to upload their data and immediately explore diverse data with similar properties, and be alerted when similar data is uploaded in future. In contrast to conventional databases which are organized by assigned metadata, *CompEngine* incentivizes data sharing by automatically connecting experimental and theoretical scientists across disciplines based on the empirical structure of the data they measure. *CompEngine*’s growing library of interdisciplinary time-series data also enables the comprehensive characterization of time-series analysis algorithms across diverse types of empirical data.

## Introduction

Taking repeated measurements of a system over time, yielding time-series data, is ubiquitous in science. Time series are studied in mathematics, statistics, and physics, and measured in disciplines ranging from biology, economics, astrophysics, and meteorology. Applications are correspondingly diverse, from high-throughput cellular phenotyping^1^, unprecedented exoplanet surveys^2^, and precision medicine, in which information is extracted from data streams ranging from heart rates to speech signals. Massive volumes of time series are also collected for diverse commercial applications, including fault identification from sensor recordings of industrial processes, fraud detection from vast streams of credit-card transactions, and marketing strategy development from the dynamics of online behaviors. The wide range of problems involving time-series data has resulted in a diversity of analysis methods, but time series and their methods are rarely compared across disciplinary boundaries^3^.

There is much to be gained from interdisciplinary collaboration on the study of time-varying systems. For example, connecting researchers studying similar real-world dynamics could prompt them to work collaboratively to understand the common patterns in their data. Similarly, connecting simulations of time-varying model systems (for which underlying mechanisms are known) to the types of real-world systems that exhibit similar dynamics could connect theoreticians and experimentalists to better understand the mechanisms underlying empirical observations. Due to large barriers to identifying commonalities, and hence areas for productive collaboration around common problems, initiating meaningful interdisciplinary connections remains a key challenge.

Here we introduce *CompEngine*, a self-organizing library of interdisciplinary time-series data that automatically highlights meaningful interdisciplinary connections between time series. *CompEngine* uses a common feature-based representation of time series to organize them according to their computed properties. *CompEngine* contains an initial set of over 24 000 time series encompassing recordings from a wide range of: empirical systems, including birdsong, population dynamics, electrocardiograms (ECG), heart-rate intervals, gait, audio, finance, meteorological, and astrophysical data; and synthetic model systems, including data generated from simulating sets of differential equations, iterative maps, and stochastic processes^3^. Each time series is annotated with user-provided metadata about what system was measured and how it was recorded or simulated.

As users upload and share their own data, connections between different data objects are updated automatically as the library grows and reorganizes itself. This process often yields surprising interdisciplinary connections between the properties of empirical data generated from real-world systems and synthetic model-generated data, thus lowering the barrier for fruitful interdisciplinary collaboration by connecting scientists through the data they analyze. *CompEngine*’s large library of time-series data can be downloaded and used as a representative interdisciplinary data resource to characterize the performance of new time-series analysis algorithms on diverse empirical data. In this paper, we motivate and introduce *CompEngine*, describe the research underlying its functional machinery, and explain how we envisage it being of broad utility for the scientific time-series analysis community.

## Approach

For a self-organizing library to meaningfully structure diverse data, it requires an appropriate measure of similarity between pairs of objects in the library. The fundamental data object in *CompEngine* is a univariate and uniformly-sampled time series (or, generally, any data that can be represented as an ordered vector of real numbers^4^). Our challenge is therefore to develop a similarity measure that can compare time series measured at different sampling rates, from different systems, and for different durations of time. Drawing on previous research, we achieve this using a feature-based representation^3–6^.

### Feature-based representations of time series

There are myriad ways two time series can be compared^4^, but computing a set of features from the measured dynamics allows a time series to be represented as a point in a common feature space, regardless of how/where it was measured. Such ‘feature-based’ representations of time series have been used to successfully tackle a wide range of problems^4^, including classification (or regression)^3,5,7^, clustering^8^, forecasting^9^, and anomaly detection^10^. To generate a feature vector, a univariate time series of *T* ordered measurements, *x*_*t*_ (*t* = 1, 2, …, *T*), is mapped to a set of *F* features, *f*_*i*_ (*i* = 1, 2, …, *F*). Each feature is the real-valued output of some algorithm applied to the time series. Features capture different types of statistical properties of time series, such as their: distribution of values (regardless of their sequential ordering); autocorrelation (how time-series values are correlated to themselves through time); stationarity (how statistical properties of a time series vary across a recording); complexity and predictability (e.g., quantified using tools from information theory); or some characteristic of a fitted time-series model (their parameters and goodness of fit)^4^. The feature-based distance between two time series, **x**^(*j*)^ and **x**^(*k*)^, is then defined in terms of the distance between the feature vectors, **f**^(*j*)^ and **f**^(*k*)^, as *d*[**f**^(*j*)^, **f**^(*k*)^], from some distance metric, *d* (e.g., Euclidean). To weight all features equally in the distance calculation, the values of each feature are normalized across the time-series dataset; here we use an outlier-robust sigmoidal normalization^3^. Our core problem then becomes how to define an *F*-dimensional feature space: **x** → **f** ∈ ${{\mathbb{R}}}^{2}$, in which feature-based distances capture meaningful differences between the vastly different types of empirical dynamics contained in a general time-series data library like *CompEngine*.

### Using feature-based similarity to structure diverse data

A feature-based representation enables diverse time series to be compared meaningfully, but how do we select which features to use, given the vast interdisciplinary literature on time-series analysis^4^? Recent work introduced an approach known as ‘highly comparative time-series analysis’^3,4,7^. This approach is implemented in the software package, *hctsa*^5^, which includes algorithmic implementations of over 7000 time-series features. To investigate whether such a feature-based representation can organize different types of dynamics, we used *hctsa* to generate feature-based representations of data from fifteen different classes that encompass both: (i) simulated data, from deterministic dynamical systems, discrete iterative maps, stochastic differential equations, and random noise series; and (ii) empirical data, from seismology, river flow, share prices, log returns of financial series, ionosophere fluctuations, sound effects, animal sounds, music, electrocardiograms, heart-rate (RR) intervals, and gait dynamics. The dataset is described in detail in Supplementary Information and data and code to reproduce this analysis is also provided.

**Fig. 1:**
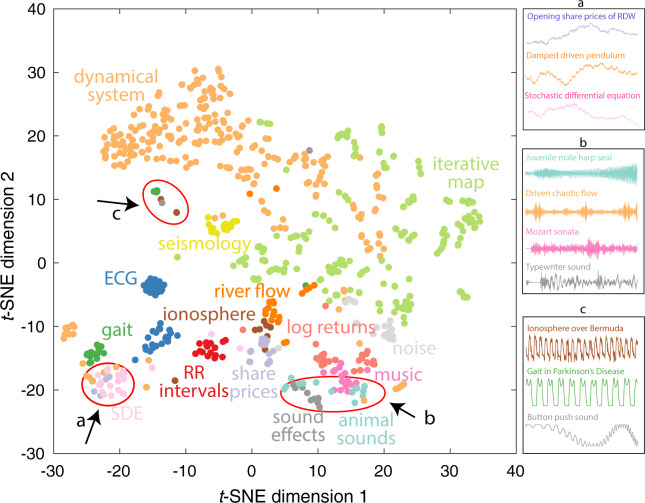
Diverse time series (of varying types, sampling rates, and durations) are organized meaningfully in a common feature space. We plot each time series in a reduced, two-dimensional feature space, computed with *t*-SNE^11^ from a high-dimensional feature space. Each time series is labeled according to a set of broad categories using color (see Supplementary Information for descriptions of each category). Most categories occupy distinct parts of the space, and categories of data with similar dynamical properties are close in the space. Interesting connections between distinct systems are flagged where distinct classes of overlap; three examples are annotated (as ‘a’, ‘b’, and ‘c’), and some examples of time series in each area are visualized as 1000-sample segments in the right panels of the figure.

Figure 1 shows the two-dimensional *t*-SNE projection^11^ of this diverse dataset from the full (>7277-dimensional) *hctsa* feature space. The data-driven organization meaningfully represents the structure of the dataset, with distinct categories of data occupying characteristic parts of the space. For example, the lower-right part of the space, labeled ‘b’, contains audio data: clusters of sound effects, animal sounds, and music. As shown in Fig. 1b, time series in this region of the feature space are visually similar, including male juvenile harp seal audio, a downsampled excerpt from a Mozart sonata, the sound of a typewriter, and a numerically simulated chaotic flow that captures many of the oscillatory and bursty properties of real audio data ($\ddot{x}$ = −*x*^3^ + sin(Ω*t*), Ω = 1.88^12^). Other examples are annotated, including the slow fluctuations of time series in region ‘a’, which contains opening share prices, a simulated damped driven pendulum, and the output from a stochastic differential equation (SDE). Periodic dynamics are concentrated in region ‘c’, including gait dynamics of patients with Parkinson’s disease, ionosphere measurements, and the audio of a button-push sound effect. Overlaps between the structure of real-world dynamics and that of time-varying model systems highlight fruitful possible connections between theory and experiment. For example, the area labeled ‘a’ in Fig. 1 contains data generated from stochastic differential equations (‘SDE’, pink) and share-price time series (purple). The types of geometric Brownian motion SDEs in this area of feature space exhibit similar stochastic dynamics to that of share prices, and are indeed commonly used to model financial time series. Additional examples of informative data-driven matches between empirical and model dynamics, including for rainfall patterns, astrophysical recordings, and human speech have been described previously^3^. This ability to make meaningful connections between diverse types of time series through a common feature space representation forms the basis for *CompEngine* being an informative self-organizing library of time-series data.

**Fig. 2:**
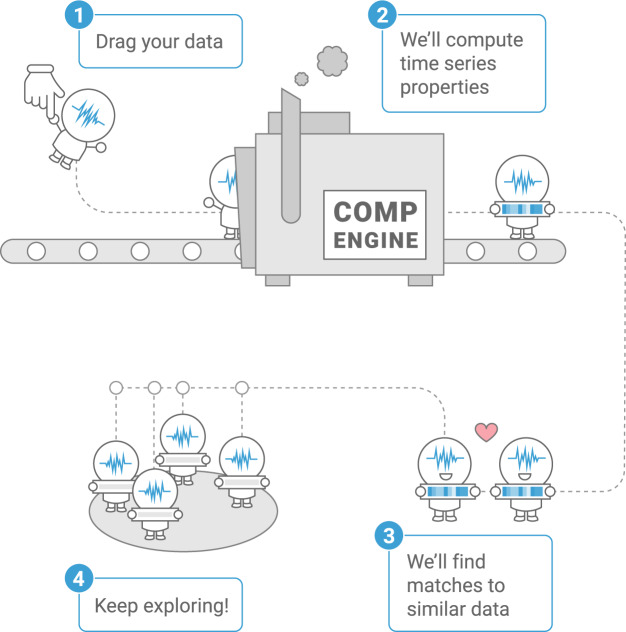
Using *CompEngine* to understand interdisciplinary connections between time series. After uploading a time series (Step 1), we compute a set of features (Step 2) which are used to calculate a similarity score between the new data and all existing data in the library (Step 3). The data context can then be explored through an interactive visualization and the data can be contributed to grow the library.

**Fig. 3:**
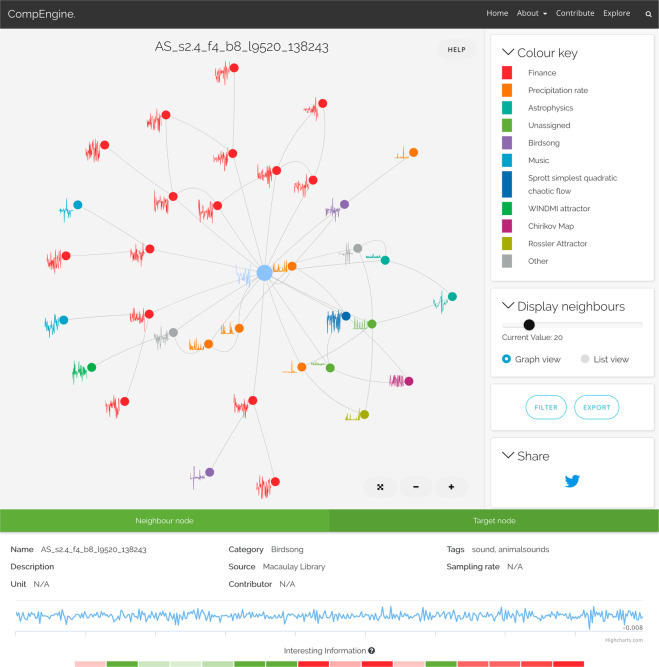
*CompEngine* allows users to upload a time series and automatically find and visualize connections to similar types of empirical and model-generated time-series data. Here we show *CompEngine*’s web-based interface for visualizing similar types of time-series data as an interactive network. The target time series—in this example birdsong from the black-bellied plover—is shown as the central light blue node, with neighbors plotted around it, colored according to their categorical assignment (labeled in the ‘Colour key’). Neighbors range from other types of audio, e.g., birdsong (purple) and music (blue), as well as data generated from time-series models, e.g., the WINDMI attractor (forest green) and the Chirikov Map (violet). Information about the target node (or a neighboring node of interest) is shown in the lower panel, which includes metadata, an interactive time-series visualization, and computed values of the time-series features. Feature values are visualized using a traffic-light color scheme, highlighting time-series properties that are exceptionally high (green) or low (red) compared to the rest of the *CompEngine* library (information is shown on hover).

### A canonical time-series feature vector

Using *hctsa* to convert time series to comprehensive feature vectors requires the computationally expensive step of calculating over 7000 features. For an efficient online implementation as *CompEngine*, we require a more computationally efficient reduced feature set. In recent work, the full *hctsa* feature library was reduced to a smaller subset of interpretable features that show high classification performance and exhibit minimal redundancy across a wide range of classification tasks, yielding a set of 22 features called *catch22*^6^. These features are implemented efficiently in C and capture the conceptual diversity present in *hctsa*, incorporating measures of autocorrelation, predictability, stationarity, distribution of values, and self-affine scaling^6^. Compared to the full *hctsa* feature set, distances between pairs of time series are highly correlated in the *catch22* feature space, *r* = 0.77 (using the time series analyzed above, cf. Fig. 1). Thus, *catch22* structures time-series datasets in a similar way to the full set of *hctsa* features, but at a small fraction of the computational cost (and without requiring a software license), making it a feasible and scalable solution for a self-organizing library like *CompEngine*.

## Functionality

Above, we showed how representing time series in a common feature space provides the basis for building a self-organizing library of time-series data. A comprehensive feature library, *hctsa*, can be reduced to a smaller number of efficiently coded features (e.g., as *catch22*) to facilitate faster feature computation and therefore data comparison. In this section, we describe our implementation of an interactive time-series data library as *CompEngine* that self-organizes using computed features of the uploaded data.

### Retrieving an interdisciplinary context for time series

Figure 2 illustrates the basic pipeline through which a user uploads their time series and visualizes connections to similar data in the *CompEngine* library.

#### Data upload

Upload of time-series data supports text files (.txt, .csv) and Excel files (.xls, .xlsx) containing a single column vector of real numbers. Audio data upload is also supported (.mp3 or .wav; the audio is converted to a time series using the first audio channel with floating-point encoding and a minimum sampling rate of 4 kHz). All uploaded data is licensed under the ‘no rights reserved’ CC0 license. For computational efficiency, individual time series containing more than 10 000 samples are truncated to the first 10 000 samples. *CompEngine* also supports upload of multiple univariate time series through a bulk upload function.

Uploaded data can be permanently added to the *CompEngine* library by providing basic metadata that is sufficient to allow new users to be able to understand it. This includes: *Name*, *Sampling Rate*, and *Description*, as well as: *Source* (identifies who/where/how the data were measured), *Category* (a hierarchical categorization of time-varying synthetic and real-world systems), and *Tags* (any additional keywords). Users may select from an existing Category, or suggest a new one, specifying a parent Category to place the proposed Category in the hierarchy. Tags are unrestricted and allow users to set useful, machine-readable and easily searchable keywords to their data. To facilitate automatic connections to new data, users can optionally choose to provide contact information and opt-in to regular updates when new data is uploaded to *CompEngine* in the future that is similar to the uploaded time series.

To maintain the quality of the database, all new individual time-series uploads are added to *CompEngine* only after being approved by an administrator. New proposed Categories also require administrator approval. Greater care is given to bulk time-series uploads, which require prior approval via a bulk upload request.

#### Interactive visualization of similar time series

As shown in Fig. 3, *CompEngine* provides an interactive network visualization of the nearest neighbors to a target time series. These nearest neighbors are those with the minimal feature-based Euclidean distances to the target time series. Each node corresponds to a time series, and is colored by its category label and annotated with a representative time trace. Links in the network capture the feature-vector similarity between pairs of time series. Users can interactively zoom in or out of the network and filter on specific sets of the categories of retrieved neighbors and can share the results (to Twitter or as a URL). There is also the option to use a ‘List view’ that shows the nearest neighbors as a table sorted by similarity that includes an interactive visualization of each matching time series. Users can use their domain knowledge to investigate interesting connections between their data and these most similar matches that exist in the *CompEngine* library.

Detailed inspection of time traces and metadata of neighboring nodes can be done using the inspector panel (at the bottom of the interface in Fig. 3). Double-clicking on a neighboring time series allows the user to go to the neighborhood of that time series, allowing progressive exploration of the database from one local neighborhood to the next. Users can also access basic information about the canonical feature vector of their data to understand where their data is placed with respect to other data in the library for each feature, flagging cases where the data is exceptional, e.g., ‘the lag-9 autocorrelation of this time series is in the top 1% of data in our library’.

The interactive online visualization provides useful insights into individual time series and their nearest matches, but some users may wish to perform more sophisticated analyses on the matching data (such as investigating the types of features that drive different types of clustering patterns). To facilitate this, *CompEngine* allows users to export all matching data as a .json or a compressed (.zip) directory of .csv files.

As well as new, uploaded data, users can also interactively explore the existing data library, as organized by Source, Category, and Tag, or by a custom search.

### Data download

Data can be download from *CompEngine* as:

1. An individual time series (through ‘download’ button associated with every time series),
2. Multiple time series matching a given search criterion (through ‘download all on page’ button), or
3. The full time-series database as a bulk download.

In all cases, data can be downloaded in either compressed .csv (.zip) or .json format. *CompEngine* also includes a public API that allows users to retrieve individual time series, or sets of time series that match specified criteria (e.g., by labeled category). This allows researchers to programmatically query *CompEngine*, providing immediate access to the latest time-series data library.

## Utility

*CompEngine* makes a large library of diverse time-series data freely available to the public, as well as interactive tools to aid exploring the library. By allowing the library to grow over time through community contributions, it can provide a good sample of the type of time-series data studied across different scientific applications. In the context of time-series analysis, in this section we describe examples of new types of science enabled by *CompEngine*.

### Connecting scientists through their data

A self-organizing library structures data objects empirically according to their dynamical properties, not their assigned metadata. As described above, and shown in Fig. 1, this can highlight unexpected connections, including: (i) between real-world and simulated data—suggests relevant mechanistic or statistical models for a real-world system; and (ii) between empirical dynamics of two real-world systems—highlights new opportunities to collaborate across interdisciplinary borders to understand connections between seemingly disparate systems. *CompEngine* provides a ‘Contact Contributor’ button for time series that have been contributed by a user who has provided contact information, allowing users to connect through similarities in their data. *CompEngine* also continues to search for new matches as additional data is uploaded in the future, and can alert the user (by email) to future matches as they occur. By treating theoretical and diverse types of empirical time series in the same way, *CompEngine* thus provides a direct incentive to data sharing: the user learns more about how their system of interest relates to other synthetic and real-world systems, both immediately at the time of upload, and into the future as the time-series data library evolves.

### Diverse data for evaluating analysis algorithms

In practice, the selection of a time-series algorithm for a given application is based on the subjective experience of the data analyst. Moving towards a more systematic procedure requires a comprehensive understanding of the characteristics of the data that a given algorithm performs well on^13^, given that no single algorithm can exhibit strong performance on all types of data^14^. *CompEngine* provides access to a large and growing data repository to facilitate the evaluation of analysis algorithms on diverse time-series data, allowing us to comprehensively and objectively understand the strengths and weaknesses of different time-series analysis algorithms applied to different types of data. This process may indeed highlight unexpected examples of datasets for which the new method performs well on, inspiring new interdisciplinary collaborations on common problems. *CompEngine* can thus be used to understand the strengths and weaknesses of different time-series analysis algorithms on different applications, contrasting the common practice of evaluating new algorithms on manually selected data (which involves selection bias). By fingerprinting the usefulness of different algorithms on different types of data, future methods development could be empirically tailored to the types of problems that our current analysis toolbox performs poorly on.

### A template for other self-organizing data libraries

*CompEngine* uses the example of time series to demonstrate how complex data objects can be projected in a common feature space and organized on the basis of their empirical properties. The benefits of such a self-organizing library are not just applicable to time-series analysis, but could also be extended to other data objects, including complex networks^15^, images, point clouds, and multivariate classification datasets^16^. We hope that *CompEngine* may serve as a template for developing new, self-organizing libraries of other data types that encourage broader scientific collaboration on common data.

## Summary

While recent years have seen dramatic growth in data sharing, including in scientific research^17,18^, data repositories are typically organized only on the basis of user-assigned metadata. *CompEngine* adds a computational layer of extracted features to self-organize a large repository of time-series data, automatically retrieving interesting connections between diverse time series. To our knowledge, the platform is the first self-organizing collection of scientific data, containing an initial library of over 24 000 diverse time series. Compared to conventional data repositories, this provides a direct incentive for data sharing, with users immediately obtaining new understanding of the interdisciplinary context surrounding their data, and an option to be notified when similar data are uploaded in the future. This resource is relevant to many applications, from individuals self-recording their heart rhythms or sleep patterns through a wearable device, to those probing a portfolio of assets or examining drilling profiles. We envisage *CompEngine* becoming a unifying portal that links disparate users—be they scientists or data analysts—who currently work isolated from one another due to high barriers to comparison, and hence collaboration.

## Supplementary information

Supplementary Information

## Data Availability

As described in this article, the data analysed in the current study is available at www.comp-engine.org, and will update in real time as new data is contributed. Data (and computed features) used for the case study (Fig. 1) is available as the *Empirical 1000* time-series dataset at 10.4225/03/59c88e1e51868^19^.

## Code availability

Matlab code for reproducing the case-study analysis of diverse empirical time-series data is available at https://github.com/benfulcher/Empirical1000_LowDimProj. Source code for the web implementation of the CompEngine interface is available at https://github.com/compenginecode/compengine-frontend. Source code for the API for querying the CompEngine database is available at https://github.com/compenginecode/compengine-api.
