# Supplementary material for: A self-organizing, living library of time-series data
Source: Sci Data. 2020 Jul 7;7:213. doi: 10.1038/s41597-020-0553-0 (PMC7341818; doi:10.1038/s41597-020-0553-0)
Supplement: Supplementary file 1 — Supplementary Information [file 41597_2020_553_MOESM1_ESM.pdf]

Supplementary Text for 'A self-organizing, living library of time-series data'

Ben D. Fulcher[1*], Carl H. Lubba[2], Sarab S. Sethi[2], and Nick S. Jones[2]

[1]School of Physics, The University of Sydney, Sydney, NSW, 2006, Australia [2]Mathematics Department, Imperial College London, Huxley Building, Queen's Gate, London SW7 2AZ, UK. *Corresponding authors: Ben Fulcher (ben.fulcher@sydney.edu.au) and Nick Jones (nick.jones@imperial.ac.uk).

Low-dimensional projection of diverse data

To demonstrate the utility of a feature-based representation of diverse time series, we investigated the structure of a low-dimensional projection of a high-dimension feature space computed using *hctsa*[1]. All data and code to reproduce this analysis are available via `https://github.com/benfulcher/Empirical1000_LowDimProj`. Features were normalized according to a scaled sigmoidal transformation[2], filtering out columns with any bad values, resulting in a data matrix of dimensions 786×5131 (time series $\times$ features). The dimensionality reduction was computed using t-SNE[3], after reducing the data matrix to a space of 50 principal components, and using the barnes-hut approximation for t-SNE (implemented in Matlab as `tsne`). Our example included a subset of data classes taken from a set of 1000 empirical time series[4]. We restricted the analysis to data labeled into the following 15 groups: 'dynamical system': 274 time series simulated numerically from diverse systems of ordinary differential equations (ODEs); 'iterative map': 221 time series simulated numerically from diverse iterative maps[5]; 'SDE': 23 time series simulated numerically from stochastic differential equations; 'noise': 20 time series of uncorrelated random noise taken from distributions including beta, binomial, and normal; 'seismology': 16 time series taken from a dataset of earthquakes and explosions; 'ionosphere': 13 recordings from the Earth's ionosphere, taken from SPIDR[6]; 'ECG': 40 ECG time series from Physionet (varying in length from 1200 to 10 000 samples)[7]; 'gait': 20 gait time series, 19 of which are from Physionet[7]; 'RR intervals': 16 RR interval series (varying in length from 1900 to 8600 samples)[7]; 'music': 17 time series taken from downsampled audio recordings of music in styles ranging from Baroque through to post-metal; 'sound effects': 12 time series taken from audio recordings of various sound effects, including a doorbell sound, a food processor, and urination,; 'animal sounds': 14 time series taken from the Macauley Library (`https://www.macaulaylibrary.org/`), including sounds from the Harp Seal, Marbled Wood-Quail, and the Red Junglefowl; 'share prices': 19 share price

time series from Yahoo Finance; and 'log returns': log-return transformations of financial time series.

References

[1] B. D. Fulcher and N. S. Jones. *hctsa*: A Computational Framework for Automated Time-Series Phenotyping Using Massive Feature Extraction. *Cell Sys.* **5**, 527 (2017).

[2] B. D. Fulcher, M. A. Little, and N. S. Jones. Highly comparative time-series analysis: the empirical structure of time series and their methods. *J. Roy. Soc. Interface* **10**, 20130048 (2013).

[3] L. v. d. Maaten and G. Hinton. Visualizing data using *t*-SNE. *J. Mach. Learn. Res.* **9**, 2579 (2008).

[4] B. D. Fulcher. 1000 Empirical Time series. *figshare* `https://doi.org/10.4225/03/59c88e1e51868` (2017).

[5] J. C. Sprott. *Chaos and time-series analysis.* Oxford University Press (2003).

[6] M. Zhizhin, E. Kihn, R. Redmon, D. Medvedev, and D. Mishin. Space physics interactive data resource—SPIDR. *Earth Sci. Informat.* **1**, 79 (2008).

[7] G. B. Moody, R. G. Mark, and A. L. Goldberger. Physionet: a web-based resource for the study of physiologic signals. *IEEE Eng. Med. Biol.* **20**, 70 (2001).
